# Survival outcomes of patients with advanced oral cavity squamous cell carcinoma treated with multimodal therapy: a multi-institutional analysis

**DOI:** 10.1186/1916-0216-42-30

**Published:** 2013-04-19

**Authors:** Han Zhang, Peter T Dziegielewski, Vince L Biron, Jacek Szudek, Khaled H Al-Qahatani, Daniel A O’Connell, Jeffrey R Harris, Hadi Seikaly

**Affiliations:** 1 Division of Otolaryngology-Head and Neck Surgery, University of Alberta, Edmonton, Alberta, Canada; 2 Department of Otolaryngology-Head and Neck Surgery, King Saud University, Riyadh, Saudi Arabia

## Abstract

**Background:**

The oral cavity is the most common site for head and neck squamous cell carcinoma. Treatment of advanced stage oral cavity squamous cell carcinoma (OCSCC) has classically involved surgical resection with postoperative adjuvant radiotherapy (S-RT).Despite this aggressive dual modality therapy, the disease outcomes have remained poor. The treatment options expanded in 2004 when two international trials showed the addition of postoperative chemotherapy to radiation improved outcomes. These trials were, however not oral cavity site specific.

**Objective:**

To assess survival outcomes of advanced OCSCC treated by differing modalities. The primary goal was to determine if the addition of postoperative chemotherapy (S-CRT) improves survival compared to other treatment regimens.

**Methods:**

Demographic, pathologic, treatment, and survival data was obtained from patients diagnosed with OCSCC from 1998–2010 in Alberta, Canada. 222 patients were included in the final analysis from 895 OCSCC patients. Actuarial overall, disease-specific, disease-free, and metastasis-free survivals were estimated with Kaplan-Meier and Cox regression analyses. Patients were grouped by treatment.

**Results:**

Patients receiving S-CRT had improved overall, disease-specific, disease-free, and metastasis-free survival compared to S-RT, CRT or RT(p < 0.05). Two and five year estimated overall survival was significantly higher in the S-CRT group at 77 and 58% (p < 0.05), versus S-RT with 55 and 40% rates(p < 0.05). Results were similar for disease-specific, disease-free, and metastasis free survival with S-CRT being favoured. Patients with extracapsular spread (ECS) treated with S-CRT versus S-RT had 55% survival advantage at 5 years (p < 0.05).

**Conclusion:**

This study shows that adding adjuvant chemotherapy to S-RT improves survival outcomes in advanced OCSCC, especially in patients with ECS.

## Introduction

The oral cavity is the most common site for head and neck squamous cell carcinoma. Over 3,000 new cases will be diagnosed in Canada in 2012 [1] with nearly half of these patients presenting with stage III/IV or advanced stage disease. Treatment of advanced stage oral cavity squamous cell carcinoma (OCSCC) has classically involved surgical resection with postoperative adjuvant radiotherapy (S-RT) [2]. Despite this aggressive dual modality therapy, the disease outcomes have remained constant at 30% local or regional disease recurrence, 25% distal metastases, and a 40% five-year survival.

The treatment options expanded in 2004 when level I evidence was established with the findings of the Radiation Therapy Oncology Group (RTOG) 9501 and European Organization for Research and Treatment of Cancer (EORTC) 22931 trials [3,4]. These two large-scale, independent, but similar, trials conducted in the U.S. and Europe demonstrated that compared to postoperative radiotherapy (RT) alone, adjuvant concurrent chemo-radiotherapy (CRT) for advanced stage OCSCC was more efficacious in local and regional control as well as disease-free survival [5-9]. Based in part on these landmark trials, many centers today have adopted triple modality therapy consisting of surgery and adjuvant concurrent chemotherapy and RT (S-CRT) for advanced stage OCSCC.

The basis of adding concurrent chemotherapy to adjuvant RT is that advanced tumors respond better to concurrent CRT rather than to RT alone [10-14]. While the two trials mentioned above provided strong evidence on the efficacy of S-CRT, they were not oral cavity site specific. The objective of this study was to assess survival outcomes of advanced OCSCC treated by differing modalities. The primary goal was to determine if S-CRT improves survival over all other treatment regimens. The secondary goals was to determine if any subgroups of advanced OCSCC patients with adverse features would receive increased benefit from the addition of chemotherapy to S-RT.

## Methods

Ethics approval for the study was granted by the University of Alberta’s Health Research Ethics Board (HREB) and the Alberta Cancer Board. The study was performed at tertiary care academic cancer centres: the Cross Cancer Institute in Edmonton, Alberta, and the Tom Baker Cancer Centre in Calgary, Alberta.

### Patients

Inclusion criteria were defined as:

1. Biopsy-proven OCSCC.

2. Treatment in Alberta with curative intent

Exclusion criteria were defined as

1. Previous oral cavity cancer with or without treatment.

2. Refusal of prescribed treatment

3. Treatment with palliative intent

4. Patients with tumors down-staged to Stage I or II from Stage III or IV on pathology

5. Incomplete data sets from chart review

### Data collection

Cancer surveillance data was retrieved by a clinical data analyst from Alberta Health Services. Data for all patients diagnosed with OCSCC in Alberta between January 1, 1998 and January 1, 2010 were obtained from the Alberta Cancer Registry (ACR). The ACR, established in 1942, is a population-based registry that records and maintains data of all new cancer cases, their treatments, and resulting deaths occurring in the province. The ACR is operated by Alberta Health Services Cancer Care and follows patients longitudinally and prospectively [15].

Demographic, survival and pathologic data were extracted from the ACR database. A physical review of outpatient, inpatient, and cancer clinic records was undertaken to confirm data accuracy and extract relevant patient, tumour, treatment, follow-up, as well as survival data. Age adjusted Charlson Comorbidity Index (CCI) scores [16], which were not included in the ACR database, were calculated using relevant comorbidities taken from chart review. Inclusion criteria followed by exclusion criteria were then applied to each patient’s data within the database to create a final data set for analysis.

### Staging

Staging of the tumours was clinical and according to the seventh edition of the American Joint Committee on Cancer (AJCC) TNM staging manual (2009) [17].

### Treatment groups

A total of four treatment groups were analyzed: 1) S-CRT, 2) S-RT, 3) CRT, and 4) RT. Group 1 patients underwent surgical resection and post-operative adjuvant CRT within 6–8 weeks of their operation. Group 2 patients received surgical resection similar to those patients in group 1 followed by adjuvant RT within 6–8 weeks post-operatively. Surgical resections consisted of tumor ablation with variations of primary closure, locoregional or free tissue transfer reconstruction, and uni- or bilateral neck dissection. Group 3 patients were treated by CRT and group 4 patients received primary RT. Patients receiving CRT therapy for metastases or palliation were not included. Failed CRT or RT patients that had salvage surgery were analyzed in an intent to treat manner as part of their original group. Doses for curative RT ranged from 6300 to 8000 Gy and for adjuvant RT from 5500 to 7000 GY. Cisplatin or carboplatin based CRT protocols were used exclusively for all patients.

Patients undergoing primary surgery for advanced OSCCC were offered adjuvant treatment if their pathologic staging remained stage III or IV. Those with adverse features on pathology were also offered chemotherapy. Such features were defined to be ECS, LVI, and PNI. Prior to 2005, adjuvant RT was offered for all patients with any or all adverse features, while after 2005, patients with one or more of these features were recommended for adjuvant CRT. Those with adverse features, after 2005, who did not receive adjuvant CRT either declined the chemotherapy or were deemed to medically frail to attempt adding chemotherapy to their planned adjuvant RT. Patients who were selected for primary RT treatment either declined surgical treatment or were found to be medically unfit for a prolonged operation.

### Outcomes

The primary outcome measure was set as overall survival. This was calculated as the time from the first date of treatment to the date of death or last known date the patient was alive. Secondary outcomes included disease-specific, disease-free, and metastases-free survival as per treatment group. Disease-specific survival was defined as the time from the first day of treatment to death as a result of the disease. Death caused by the primary cancer was therefore considered to be disease specific death. Disease-free survival was calculated from the first day of treatment to the date of disease recurrence (anywhere in the body). Thus, if patients died without any evidence of disease, they were considered disease free at the time of death. Metastasis-free survival was defined as the time from the first day of treatment to the date of distant metastasis detection.

### Follow-up

All patients were followed at regional cancer treatment centers at regular intervals following treatment. Dates of follow-up up to March 1, 2012 were recorded. Patients who were suspected of disease recurrence had lesion biopsy as well as metastatic workup. Salvage surgical resection was offered to all patients with failed induction RT and CRT treatments.

### Statistical analysis

Baseline characteristics were compared using standard modes of comparison between multiple groups. Continuous data was analyzed using ANOVA, with a Bonferoni correction factor for multiple comparisons. Categorical data was compared using the chi-squared test. Overall, disease-specific, disease-free and metastases-free survival analyses were performed using Kaplan-Meier analyses to determine year-specific estimated actuarial survival rates. The log-rank test was employed to determine the presence of significant differences (p < 0.05) between different treatment groups. Cox regression analysis, with covariates of age, gender, CCI, T stage, and N stage, was performed for overall, disease-specific, disease-free, and metastasis-free survival. Patients were grouped by treatment. Analyses were performed with SPSS Statistics 19.0 (SPSS Inc, Chicago, IL).

## Results

### Patient population

895 patients were diagnosed with OCSCC in Alberta from 1998 to 2010. Of these, 311 patients were found to have stage III or IV oral cavity tumors. 25 had palliative treatment, 15 refused any form of treatment, and 7 had therapy outside of Alberta. A total of 16 patients did not undergo planned adjuvant RT or CRT treatment with 2 patients deemed medically unfit and 14 refusing the supplemental treatment. This group was excluded from the study as they were deemed not compliant to the treatment. 12 patients that were planned to receive postoperative RT or CRT had metastasis or a second primary cancer during or prior to starting adjuvant therapies. They were excluded because their goal of treatment had become palliative since the discovery of distant metastasis or the second cancer. 6 patients with only surgical resection were excluded owing to down-staging based on their pathology report. 3 had misclassified tumours, and 5 had incomplete data. As a result, 222 patients were included in the final analysis.

Table 1 demonstrates patient demographic and tumour variables. Two hundred and twenty two patients were staged according to the seventh edition of the AJCC cancer staging manual [17]. All patients included for analysis had undergone a standard metastatic workup and all were staged M0 at the time of diagnosis. All patients were treated with curative intent. Age, gender, CCI score, N-stage, and adverse pathological features were all found to be statistically different (p < 0.05) while T-stage, overall stage, and cause of death did not demonstrate statistical difference between the treatment groups. When the two treatment groups of S-CRT and S-RT were compared there were no statistically significant differences between any of the demographic data categories.

**Table 1:** Characteristics of patients and tumors by treatment group

	**Group**				
**Variable**	**S-CRT**	**S-RT**	**CRT**	**RT**	**p-Value**
**n**	43	140	10	28	-
**Age**					
Mean, yrs	53.5	61.9	61.1	77.4	<0.001*
Range, yrs	16.3 – 78.0	35.5 – 93.6	45.2 – 74.5	53.1 – 95.0	
**Gender, no. (%)**					0.01*
Male	21 (48.8)	95 (67.9)	6 (60.0)	11 (39.3)	
Female	22 (51.2)	45 (32.1)	4 (40.0)	17 (60.7)	
**Charlston Comorbidity Index, mean score (range)**	1.9 (0–9)	2.3 (0–7)	3.0 (0–7)	5.4 (0–9)	<0.001*
**Site of tumor, no. (%)**					
Tongue,	23 (53.5)	51 (36.4)	6 (60)	8 (28.6)	0.92
Floor of Mouth	8 (18.6)	37 (26.4)	2 (20.0)	7 (25.0)	
Other	12 (27.9)	52 (37.1)	2 (20)	13 (46.4)	
**cT-stage, no. (%)**					0.16
1	1 (2.3)	9 (6.4)	1 (10.0)	0 (0.0)	
2	15 (34.9)	39 (27.9)	2 (20.0	5 (17.8)	
3	13 (30.2)	28 (20.0)	1 (10.0)	4 (14.2)	
4	14 (32.6)	64 (45.7)	6 (60.0)	19 (67.8)	
**cN-stage, no. (%)**					<0.001*
N0	2 (4.7)	39 (27.9)	2 (20.0)	12 (42.9)	
N1	4 (9.3)	52 (37.1)	3 (30.0)	11 (39.3)	
N2a	3 (7.0)	23 (16.4)	2 (20.0)	2 (7.1)	
N2b	18 (41.9)	15 (10.7)	2 (20.0)	2 (7.1)	
N2c	14 (32.6)	7 (5.0)	0 (0.0)	1 (3.6)	
N3	2 (4.7)	4 (2.9)	1 (10.0)	0 (0.0)	
**Perineural Invasion, no. (%)**	21 (48.8)	34 (24.3)	/	/	0.004*
**Lymph-vascular Invasion, no. (%)**	31 (72.1)	24 (17.1)	/	/	<0.001*
**Extracapsular spread, no. (%)**	27 (62.8)	46 (32.9)	/	/	0.001*
**Overall stage, no. (%)**					0.60
III	4 (9.3)	46 (32.9)	0 (0)	7 (25)	
IV	39 (90.7)	94 (67.2)	10 (100)	21 (75)	
**Death, no. (%)**	17 (39.5)	89 (63.6)	10 (100)	26 (92.9)	
**Death, cause (%)**					0.37
Primary Cancer	47.1	85.4	90	92.3	
Other Cancer	23.5	5.6	0	3.8	
Non-Cancer	29.4	10.1	10	11.5	

Abbreviations: *S*-*CRT*, Surgery with adjuvant chemo-radiation therapy; *S*-*RT*, Surgery with adjuvant radiation therapy; *CRT*, Primary chemo-radiation therapy; *RT*, Primary radiation therapy; *no*., Number; *yrs*, Years; *cT*-*stage*, Clinical T-stage; *cN*-*stage*, Clinical N-stage.

* There were no statistically significant differences between the two treatment groups of S-CRT and S-RT.

Table 2 demonstrates the measure of loco-regional recurrence by treatment groups. The S-CRT group showed a 21% lower recurrence rate compared to S-RT. CRT and RT had much higher recurrence rates.

**Table 2:** Measure of loco-regional recurrence by treatment group

**Groups**				
**Variable**	**S-CRT**	**S-RT**	**CRT**	**RT**
**Recurrence, no. (%)**	10 (23.3)	76 (54.3)	7 (70)	11 (39.3)
**No recurrence, no. (%)**	33 (76.7)	64 (45.7)	3 (30)	17 (60.7)

Abbreviations: *S*-*CRT*, Surgery with adjuvant chemo-radiation therapy; *S*-*RT*, Surgery with adjuvant radiation therapy; *CRT*, Primary chemo-radiation therapy; *RT*, Primary radiation therapy; *no*., Number.

Charlson co-morbidity index (CCI) scores were grouped as per Table 1 and were calculated using the age adjusted version of the index. An age adjusted comorbidity score of 2 was associated with a 2.10 estimated relative risk while a score of 8 or higher was conferred to have a 19.37 estimated relative risk [16]. CCI is designed to stratify patients in order to control for the confounding influence of comorbid conditions on overall survival [18]. The age adjusted CCI used in this study accounts for both age and comorbidity as a predictor of death from comorbid disease.

### Survival analysis

**Figure 1:**
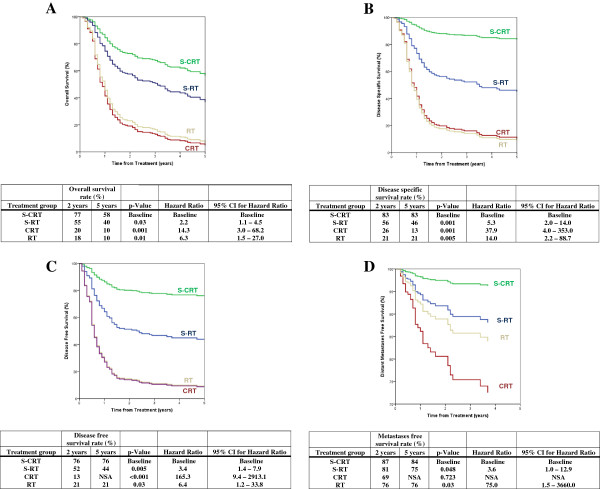
(A) Overall survival, (B) disease-specific survival, (C) disease-free survival, and (D) metastasis-free survival rates for patients with stage III or IV oral cavity tumours according to treatment group (Cox regression analysis).

**Figure 2:**
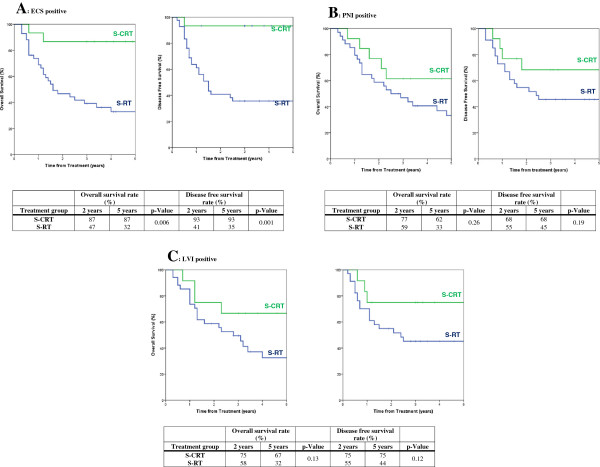
(A) ECS positive, (B) PNI positive, and (C) LVI positive overall and disease-free survival for patients with stage III or IV oral cavity tumours according to treatment group (Cox regression analysis).

The mean follow-up for all patients was 3.52 years (SD = 1.8 years, Range = 2.41 – 5.21 years). A total of 142 patients died, with 115 patients dying of OCSCC related causes. 10 died of other cancers and 17 died of non-cancer related causes. Figure 1 demonstrates Cox-regression analysis for estimated overall, disease-specific, disease-free, and metastasis-free survival by treatment group for all patients of this study. Comparisons in the Cox-regression analysis were made to the S-CRT group which served as a baseline for comparison. Figure 2 demonstrates survival results of S-CRT versus S-RT grouped by adverse feature found on pathology.

## Discussion

Despite regimens that allow for organ preservation in selected patients, surgical resection followed by postoperative RT has become the standard of care for stage III and IV OCSCC [19-21]. Even with this dual therapy, local or regional disease recurrence and low survival remain an concern [22]. Patients with adverse features of ECS, LVI, and PNI are at particular risk of disease progression and higher death rates [23]. Promising results emerged from the use of various combinations of postoperative CRT in randomized studies from 1970–1990. [24,25] Further advancement in OCSCC treatment didn’t come until 2004 with the RTOG 9501 and EORTC 22931 trials which suggested that improved disease-free survival in addition to local and regional control can be achieved with the addition of adjuvant chemotherapy to postoperative RT. The EORTC trial showed that the addition of adjuvant chemotherapy allowed for significantly increased rates of local control, disease-specific survival, and overall survival, without high incidences of late adverse effects. While improved disease-free survival, local or regional recurrence, and rates of distant metastases were shown in the RTOG trial, overall survival was not found to be superior. These studies, while being landmark trials with level I evidence, were not site-specific for oral cavity tumours and had marked differences in selection criteria [26]. A substantially higher proportion of patients in the RTOG trial had N_2-3_ disease. Both studies also did not account for confounding variables when calculating survival curves. These factors in combination with a review of the literature in 2005 for postoperative CRT confirmed the need for more evidence for adjuvant CRT therapy [26]. The current study sought to confirm the findings of the RTOG and EORTC studies, while being site specific for OCSCC.

Overall survival for stage III and IV OCSCC was found to be the highest in the S-CRT group (Figure 1). Those treated with S-RT were 2.2 times more likely to die than those treated with S-CRT and 5.3 times more likely to die secondary to disease specific causes than the S-CRT group (p < 0.05). Furthermore, patients in the S-RT were 3.4 more likely to experience disease recurrence than those treated with S-CRT (p < 0.05). This is supported by the notably lower overall loco-regional recurrence rates found in the S-CRT arm. 5-year overall survival rates (58%) for the S-CRT and the S-RT group (40%) were compble to those of the EORTC study. While disease-free survival at 2-years for patients in the S-CRT (76%) group was similar to the findings in the RTOG trial, the S-RT (52%) group did not fare as well. This discrepancy could be attributed to varied sensitivity of different head and neck sites to radiotherapy that was not septed in the RTOG trial [3].

2- and 5- year metastases-free survival showed that the S-CRT group had the highest rates at 87% and 84% respectively (Figure 1). Patients receiving S-RT were 3.6 times more likely to develop metastasis compared to S-CRT. These findings concur with the RTOG and EORTC trials in that the addition of chemotherapy to adjuvant RT improves distant metastatic control [3,4]. The 2- and 5- year overall survival for patients with ECS positive pathology was significantly higher (p < 0.05) in patients treated with S-CRT, (both at 87%) than with S-RT, found to be 47 and 32% respectively (Figure 2). PNI and LVI positive patients however did not achieve statistically significant differences in overall or disease-free survival. The S-CRT group disease-free survival at 2- and 5-year intervals was highest in the ECS group at 93 and 93%, (p < 0.05) compared to S-RT which was found to be 41 and 35% respectively. While disease-free survival was not septed by ECS status in the EORTC and RTOG trials, it was shown to significantly improve with postoperative CRT, which is congruent with our findings [3,4]. Patients with ECS also had improved disease-specific and metastasis-free survival in the S-CRT group (p < 0.05). ECS positive patients therefore seem to benefit more in overall, disease-free, disease-specific, and metastasis-free survival when treated with adjuvant CRT than with adjuvant radiotherapy alone. These results compare favorably with the results of the EORTC and RTOG trials where ECS was the only adverse feature that showed improvement in overall survival with S-CRT in both studies [26].

Standards of care are ideally based on the highest level of evidence available, which in many cases consists of randomized control trials (RCT). RCTs typically have stringent enrolment criteria and as a result, physicians must be careful in applying results to their practice. The effects of these studies are then important to be examined on the population they are applied to. Population-based studies, which are known for their external validity can then be used to externally validate an RCT [27]. This study used some prospectively collected cancer registry data combined with a retrospective review of charts to determine survival rates. Not all variables were controlled in this study owing to its population-based design. Table 1 shows that treatment groups were not completely balanced. Older patients and those with higher CCI scores tended to receive RT treatments, and those with more advanced disease tended to have received S-CRT or S-RT. These factors were accounted for as possible confounding variables in a vigorous multivariate Cox regression analysis.

Treatment protocols on CRT in addition to adjuvant CRT or RT were not clearly defined at either the Cross Cancer Institute or the Tom Baker Cancer Center. Although this implies a potential selection bias, it also establishes a need for determining optimal therapy protocols for patients requiring CRT as well as postoperative CRT or RT [28,29].

The RTOG and EROTC trials relied on Kaplan-Meier analyses between treated groups but failed to account for possible confounding variables [24]. It is well known that age, gender, TNM staging, and even performance scores have an effect on survival [30-35]. Because of this, these variables were accounted for in a multivariate Cox-regression analysis. Age adjusted CCI was also included in the multivariate analysis as age and co-morbidities have been shown to affect risk of mortality [16]. Covariates known to influence survival were included, and statistical significance was achieved.

The results of this study support the RTOG and EORTC landmark trials. The addition of postoperative chemotherapy to S-RT for advanced OCSCC appears to improve overall survival by 22% and 18% at 2 and 5 years post-treatment, respectively. Disease free and metastases free survival also benefitted from the chemotherapy addition. This suggestion mirrors previous evidence that adjuvant CRT may prevent distant spread of disease [3,4,20,21]. While ideally an RCT specific for advanced stage OCSCC should be performed, this study serves as the first population based study confirming the survival advantage of triple modality therapy treatment for advanced stage squamous cell carcinoma of the oral cavity.

## Conclusion

Advanced stage OCSCC remains a challenging disease to treat. For many years, primary ablative surgery followed by postoperative radiotherapy was the standard of care. Landmark head and neck cancer trials in 2004 however introduced the efficacy of S-CRT. This population-based cohort study demonstrates that the addition of adjuvant chemotherapy to S-RT improves survival outcomes in advanced OCSCC. Patients with ECS show the greatest survival advantage.

### Ethics approval

This study was approved by the University of Alberta’s Health Research Ethics Board.

### Consent

Written informed consent was obtained from the patient for publication of this report and any accompanying images.

## Competing interest

The authors declare that they have no competing interest.

## Authors’ contributions

HZ is responsible for study design, statistical analysis, and manuscript preption. PD was responsible for statistical analysis and manuscript preption. VB was responsible for study design. JS, KA, DO, and JH are responsible for manuscript preption. HS is the senior author and was involved in all levels of study design, data analysis and manuscript preption. The manuscript was read and approved by all the authors.

### Presentation

This study was presented by Dr. Han Zhang at the 66th Canadian Society of Otolaryngology-Head and Neck Surgery annual meeting, Poliquin Resident’s Competition in Toronto, ON on May 20–22, 2012.

### Submission

This material has never been published and is not currently under evaluation in any other peer-reviewed publication.
